# Fetal Kidney Cells Can Ameliorate Ischemic Acute Renal Failure in Rats through Their Anti-Inflammatory, Anti-Apoptotic and Anti-Oxidative Effects

**DOI:** 10.1371/journal.pone.0131057

**Published:** 2015-06-18

**Authors:** Ashwani Kumar Gupta, Sachin H Jadhav, Naresh Kumar Tripathy, Soniya Nityanand

**Affiliations:** Stem Cell Research Facility (SCRF), Department of Hematology, Sanjay Gandhi Post Graduate Institute of Medical Sciences, Raebareli Road, Lucknow, 226014, India; Universidade de Sao Paulo, BRAZIL

## Abstract

Fetal kidney cells may contain multiple populations of kidney stem cells and thus appear to be a suitable cellular therapy for the treatment of acute renal failure (ARF) but their biological characteristics and therapeutic potential have not been adequately explored. We have culture expanded fetal kidney cells derived from rat fetal kidneys, characterized them and evaluated their therapeutic effect in an ischemia reperfusion (IR) induced rat model of ARF. The fetal kidney cells grew in culture as adherent spindle shaped/polygonal cells and expressed CD29, CD44, CD73, CD90, CD105, CD24 and CD133 markers. Administration of PKH26 labeled fetal kidney cells in ARF rats resulted in a significant decrease in the levels of blood urea nitrogen, creatinine, and neutrophil gelatinase-associated lipocalin and decreased tubular necrosis in the kidney tissues (p<0.05 for all). The injected fetal kidney cells were observed to engraft around injured tubular cells, and there was increased proliferation and decreased apoptosis of tubular cells in the kidneys (p<0.05 for both). In addition, the kidney tissues of ARF rats treated with fetal kidney cells had a higher gene expression of renotropic growth factors (VEGF-A, IGF-1, BMP-7 and bFGF) and anti-inflammatory cytokine (IL10); up regulation of anti-oxidative markers (HO-1 and NQO-1); and a lower Bax/Bcl2 ratio as compared to saline treated rats (p<0.05 for all). Our data shows that culture expanded fetal kidney cells express mesenchymal and renal progenitor markers, and ameliorate ischemic ARF predominantly by their anti-apoptotic, anti-inflammatory and anti-oxidative effects.

## Introduction

Acute renal failure (ARF) is characterized by rapidly declining renal functions induced by toxic or ischemic damage of renal tubular and vascular cells with a key role of inflammation in the pathophysiology of the disease. It is a global disease increasingly affecting people of all age groups and having a high mortality rate. The disease has no curative treatment available except renal transplantation which has its own limitations and complications [1, 2]. Thus development of new therapeutic strategies is warranted for the treatment of ARF.

Cell therapy represents a potential new therapeutic approach for ARF as stem cells may simultaneously target the key manifestations of ARF including renal vascular damage and inflammation [3, 4]. Several pre-clinical animal studies have investigated the effects of different adult stem cell types including hematopoietic, mesenchymal, endothelial and kidney stem/progenitor cells in the treatment of ARF [5–8]. Further, few studies on fetal kidney cells transplantation in rodents also support the regenerative potential of these cells after renal injury [9, 10]. However, a suitable renogenic cell type to obtain a clinically relevant therapeutic effect in ARF has not yet been achieved and no cell based clinical therapy has yet been established.

We have recently shown that rat fetal heart contains mesenchymal like stem cells that exhibit rapid proliferation, multipotent differentiation potential and constitutive expression of markers of cardiovascular lineage indicating their pre-commitment towards tissue of origin and thereby a greater efficacy in cardiac regeneration than other stem cell types [11]. In a subsequent study, we have demonstrated efficacy of these fetal stem cells in cardiac regeneration in a rat model of myocardial injury [12]. Similarly, other groups have demonstrated a promising therapeutic role of fetal pancreatic, neural and liver stem cells in the treatment of diabetes, stroke and liver disease respectively, further highlighting that stem cell therapy with tissue specific fetal stem cells may be a potential approach for tissue repair/regeneration [13–15]. More recently, we have demonstrated that fetal kidney cells ameliorate cisplatin induced acute renal failure and promote renal angiogenesis in rats [16]. These studies indicate that fetal kidney may be a rich source of different stem/progenitors cells inherently committed towards different renal lineages and thus fetal kidney cells may prove to be a novel cell type for treatment of ischemic ARF. However, there is a paucity of data on characterization and therapeutic effect of fetal kidney cells in ischemic ARF.

Therefore the aim of the present study was to isolate and characterize the fetal kidney cells derived from rat fetal kidneys and to evaluate their therapeutic effect and mechanism(s) of action in an ischemia reperfusion (IR) induced rat model of ARF.

## Materials and Methods

### Animals

Sprague Dawley (SD) rats with 225–250g weight were used in the study. The animals were housed in a constant room temperature with a 12-hours constant dark-light cycle. Food and water were supplied ad libitum. All animal experimental procedures in this study were performed as per guidelines of Institutional Animal Ethics Committee and the Committee for the Purpose of Control and Supervision of Experiments on Animals (CPCSEA), India. The protocol was approved by the Committee on the Ethics of Animal Experiments of Sanjay Gandhi Post Graduate Institute of Medical Sciences, Lucknow, India.

### Isolation and culture of fetal kidney cells

The fetal kidney cells were isolated and cultured from the kidneys of SD rat fetuses obtained at gestation day 16. The kidneys, surgically removed from fetuses of 10 pregnant female rats (10–12 fetuses /animal) were minced, pooled together and digested with 1 mg/ml collagenase type-IV (Worthington Biochemical, NJ, USA) in serum free α-MEM medium for 40 min at 37°C with intermittent stirring. After washing with α-MEM, the digested tissue was cultured at 37°C in 5% CO2 in 25 cm^2^ tissue culture flasks (BD, New Jersey, USA) in complete culture medium consisting of α-MEM medium, 2 mg/ml of Glutamax (Gibco, NY, USA), 16.5% FBS (Gibco, NY, USA) and bacteriostatic level of penicillin-streptomycin (Gibco, NY, USA). After 48 hours of seeding of digested fetal kidney tissues, the culture media containing non-adherent cells was replaced. On day 3 adherent cells were harvested by trypsinization with TrypLE Express (Gibco, NY, USA) and further cultured as above. The cells of 3^rd^ passage were used in the experiment.

### Karyotyping of fetal kidney cells

Karyotypic study was carried out as described previously [11]. In brief, the fetal kidney cells were cultured for 3 days and treated with 10 μg/μl of colcemid (Sigma-Aldrich, MO, USA) for 30 min, followed by hypotonic solution of 60 mM KCl for 6 min. The cells were then fixed with methanol/acetic acid in a ratio of 3:1 and the chromosomes of 10 metaphases were counted under the microscope for karyotypic analysis.

### Flow cytometry

The fetal kidney cells were stained with following pre-conjugated or un-conjugated antibodies: CD44-fluorescein isothiocyanate (FITC) (BD Biosciences, CA, USA), CD90-FITC, CD45-phycoerythrin (PE), MHC class II-PE (all from Abcam, MA, USA) and with unconjugated CD29, VEGFR2, EpCAM (all from Abcam, MA, USA), CD73, CD24 (both from BD Biosciences, CA, USA), CD105 (Santa Cruz Biotechnology, Texas, USA), CD133 (My Biosource, CA, USA). After 30 minutes, cells incubated with un-conjugated antibodies were washed with PBS and stained with corresponding FITC-conjugated secondary antibodies. Cells stained with isotype-matched antibodies (IgG) served as controls. After staining, the cells were fixed in 4% para-formaldehyde (Sigma-Aldrich, MO, USA) and analyzed in FACS Calibur (BD Biosciences, CA, USA). The experiment was performed in three independent preparations.

### *In vitro* production of renotropic growth factors by fetal kidney cells

The fetal kidney cells were cultured in serum supplemented culture media in 48 well plate at seeding density of 2×10^4^ cells/well at 37°C in 5% CO2. After 24 hours, this culture media of the cells was replaced with serum free media and further cultured for 48 hours under same culture conditions. The cell free culture supernatant collected after 48 hours was analyzed for different growth factors using commercial immunoassay kits for vascular endothelial growth factor (VEGF), basic fibroblast growth factor (bFGF), bone morphogenetic protein (BMP)-7 (all from Abcam, MA, USA) and insulin-like growth factor (IGF)-1 (R&D Systems, MN, USA). Fresh serum free media was used as control medium.

### Fluorescent labelling of fetal kidney cells

The fetal kidney cells were labeled with PKH26, a red fluorescent cell linker (Sigma-Aldrich, MO, USA) according to manufacturer’s protocol. Briefly, 2 x 10^7^ cells were placed in a conical bottom polystyrene tube and washed with 10mL PBS. After centrifugation at 400 x g for 5 minutes, the supernatant was aspirated leaving no more than 25 μl supernatant over the pellet. The cells resuspended in 1mL of diluent C and 1 mL of 2 x 10^−6^ M of PKH26 staining reagent was added to obtain the final volume of 2mL. The cells were then incubated at room temperature for 5 min. The equal amount of 10% of FBS (2mL) was used to stop the reaction. After that cells were centrifuged at 400 x g and washed with 10 mL complete culture media for three times. Cells were resuspended at 2 x 10^6^ cells/150 μl with normal saline to inject in animals. Labelling efficiency was >95% and the cell viability was >97%, as assessed by using fluorescent microscopy and trypan blue exclusion staining, respectively.

### Administration of fetal kidney cells in rat model of ARF

SD rats (n = 12) weighing 225–250g were anaesthetised with an intra-peritoneal injection of 80 mg/kg ketamine and 10mg/kg xylazine and they were placed in a supine position on a temperature control pad and rectal temperature was maintained at 37°C. The mid-abdominal laparatomy was performed to expose the kidneys and both renal pedicles were clamped with atraumatic vascular clamps for 45 min. Reperfusion was initiated by the removal of clamps and was visually confirmed by noting subsequent blush.

After 24 hours of reperfusion, animals were randomized in to two groups viz. saline treated (n = 6) and fetal kidney cells treated (n = 6) groups. PKH26 labeled fetal kidney cells (2 x 10^6^ cells/animal) or saline (150 μl/animal) were injected via the tail vein. Sham operated rats (n = 6) were subjected to the same surgical procedure without clamping of renal pedicles. Blood was collected at 0, 24, 48, 72, and 96 hours after reperfusion for blood urea nitrogen (BUN), serum creatinine and serum neutrophil gelatinase-associated lipocalin (NGAL) analysis. Animals were sacrificed 96 hours after reperfusion and kidney tissues were collected for analysis.

### Evaluation of therapeutic effect of the culture supernatant in rat model of ARF

Therapeutic potential of culture supernatant of fetal kidney cells was evaluated in rat model of ischemia induced ARF. Animals were randomized in to 4 groups after reperfusion viz. sham operated (n = 6), fresh culture medium treated (n = 6), culture supernatant treated (n = 6) and fetal kidney cell treated (n = 6) groups. The cell free culture supernatant was prepared as per protocol previously described by Xing *et al*. with some modifications [17]. Briefly, 2 × 10^6^ fetal kidney cells were cultured with 2 ml serum free media for 48 hours in standard culture condition. After that the culture supernatant was harvested and filtered through a 0.22 μm filtration unit (Millipore, MA, USA). A dose of 200μl of fresh culture medium or culture supernatant was injected via tail vein in ARF rats each time at 24, 48 and 72 hours after reperfusion. The fetal kidney cells (2 × 10^6^ cells/animal) were administered after 24 hours of reperfusion via tail vein in the rats. Blood was collected at 0, 24, 48, 72, and 96 hours after reperfusion for blood urea nitrogen (BUN), serum creatinine and serum neutrophil gelatinase-associated lipocalin (NGAL) analysis.

### *In vivo* tracking of fetal kidney cells

To evaluate engraftment of PKH26 labeled cells in the damaged kidney, the animals were sacrificed 96 hours after reperfusion, kidney tissues were fixed in 10% of formalin and cut in to 5 μm thick sections. Kidney sections were analysed for PKH26 positive cells by fluorescent microscopy. PKH26 positive cells were counted in 10 random high power fields (HPF) per rat (40X) and data were expressed as number of PKH26 positive cells/HPF.

### Blood biochemical analysis

Renal function was assessed by measuring BUN and serum creatinine levels by Span diagnostic kits with an autoanalyser (BioSytems BTS-330) and Serum NGAL levels were estimated by Rat NGAL ELISA Kit (BioPorto Diagnostics, Denmark) according to manufacturer’s protocol.

### Real-time quantitative PCR (RQ-PCR)

The gene expression of cytokines, growth factors and anti-oxidative markers in the kidney tissues of fetal kidney cells and saline treated ARF rats was assessed by RQ-PCR. Total RNA from kidney tissues was extracted using a TRIzol reagent (Invitrogen, CA, USA). Complementary DNA was synthesized from 1μg of total RNA using first-Strand cDNA Synthesis Kit (USB, OH, USA) according to manufacturer’s instructions. RQ-PCR was performed to measure target gene expression for cytokines viz. interleukin (IL)-1β, tumor necrosis factor (TNF)-α, interferon (IFN)-γ, IL-6, IL-10, growth factors viz. viz. bFGF, BMP-7, VEGF-A, IGF-1, and anti-oxidative markers viz. glutathione reductase (GR), glutathione peroxidase (GPx) relative to β-actin gene expression using the primers listed in Table 1. Relative fold expression values were determined applying the ΔΔ cycle threshold (Ct) method [18].

**Table 1:** List of primers used for RQ-PCR analysis.

Sl. No.	Gene	Primer Sequence (5'-3')	Accession No.
1.	β -Actin	F-CCCGCGAGTACAACCTTCT / R-CGTCATCCATGGCGAACT	NM_031144.2
2.	IL-1β	F- TGTGATGAAAGACGGCACAC / R-CTTCTTCTTTGGGTATTGTTTGG	NM_031512.2
3.	TNF-α	F-GCCCAGACCCTCACACTC / R-CCACTCCAGCTGCTCCTCT	X66539.1
4.	IFN-γ	F-TTTTGCAGCTCTGCCTCAT / R-AGCATCCATGCTACTTGAGTTAAA	NM_138880.2
5.	IL-6	F-CAAGAGACTTCCAGCCAGTTGC / R-TGGCCGAGTAGACCTCATAGTGACC	NM_012589.1
6.	IL-10	F-AGTGGAGCAGGTGAAGAATGA / R-TCATGGCCTTGTAGACACCTT	NM_012854.2
7.	bFGF	F-CGACCCACACGTCAAACTAC / R-CCAGGCGTTCAAAGAAGAAA	X61697.1
8.	BMP-7	F-CCAAGAGGCACTGAGGATGG / R-TGGTGGCGTTCATGTAGGAG	NM_001191856.1
9.	IGF-1	F-GGCATTGTGGATGAGTGTTG / R-ACGTGGCATTTTCTGTTCCT	X06043.1
10.	VEGF-A	F-TGTGAATGCAGACCAAAGAAA / R-CTGAACAAGGCTCACAGTGAAT	AY702972.1
11.	GPx	F-TGCAATCAGTTCGGACATC / R-CACCTCGCACTTCTCAAACA	NM_030826.2
12.	GR	F-ATCAAGGAGAAGCGGGATG / R-GCGTAGCCGTGGATGACTT	NM_053906.2

### Histopathology and immunohistochemistry

Kidney tissues obtained at 96 hours after reperfusion from saline treated and fetal kidney cells treated rats were fixed in 10% formalin and serial sections of 5 μm were stained with hematoxylin and eosin to evaluate sequential histopathological changes. A grading score scale outlined by Jablonski et al. [19] was used for assessment of necrotic injury to proximal tubules.

For immunohistochemical analysis, 5 μm thick paraffin sections of kidneys were deparaffinized with xylene and rehydrated in a series of alcohol and water. After rehydration, tissue sections were incubated with primary antibodies: cytokeratin (CK) 19 (Thermo scientific, IL, USA) and proliferating cell nuclear antigen (PCNA; BD Biosciences, CA, USA). After overnight incubation at 4°C, sections were washed with PBS and incubated with corresponding FITC-conjugated secondary antibodies and then stained with Hoechst dye. After that kidney sections were examined for proliferating cells per HPF under fluorescence microscope (Nikon 80i, Japan). Apoptotic scores in kidney tissue sections were determined with a terminal transferase-mediated dUTP nick-end labelling (TUNEL) staining using an In Situ Cell Death Detection Kit (Roche, Mannheim, Germany). Kidney sections were deparaffinized, rehydrated, and digested with proteinase K and labeled with TUNEL reaction mixture for 1 hour at 37°C. Sections were screened for positive nuclei per HPF under a fluorescence microscope (Nikon 80i, Japan), and 10 random sections in the cortex and outer medullary were counted for every kidney under 40 X magnification.

### Western blotting

Kidney tissues of sham operated, saline treated and fetal kidney cells treated rats were homogenised in RIPA buffer containing 1 mmol/L phenylmethanesulphonyl fluoride (PMSF) and 1% protease inhibitor cocktail (Sigma-Aldrich, MO, USA). 40 mg proteins were loaded and separated on 10% sodium dodecyl sulphate polyacrylamide gel (SDS-PAGE). After electrophoresis, separated proteins were transferred to polyvinylidene difluoride (PVDF) membranes. Membranes were blocked with 5% BSA for 1 hour at room temp and incubated overnight at 4°C with appropriate dilutions of primary antibodies: nuclear factor-κB (NFκB), intercellular adhesion molecule-1 (ICAM-1), heme oxygenase-1 (HO-1), NAD(P)H quinone oxidoreductase 1 (NQO-1), cytochrome c, caspase 3 (all from Abcam, MA, USA), B-cell lymphoma 2-associated X protein (Bax) and B-cell lymphoma 2 (Bcl2) (both from Cell Signaling Technology, MA, USA). β-actin antibody (Abcam, MA, USA) was used as a loading control. After incubation, PVDF membranes were washed three times with TBS and incubated with corresponding horse raddish peroxidase-conjugated secondary antibody for 1 hour at room temperature. All proteins were detected using super signal west pico chemiluminescent substrate (Thermo scientific, IL, USA) and quantified by densitometry using the Quantity One software (Bio-Rad, CA, USA).

### Statistical analysis

Values were expressed as Mean±SEM. Data were analysed by one way-analysis of variance (ANOVA), followed by Bonferroni multiple-comparison post hoc test. The ordinal values of Jablonski scale were analysed by non-parametric Mann Whitney test. Statistical analysis was performed using GraphPad Prism Software Version 5 (GraphPad, San Diego, CA). P value of <0.05 was considered statistically significant.

## Results

### Karyotyping and characterization of fetal kidney cells

**Fig 1:**
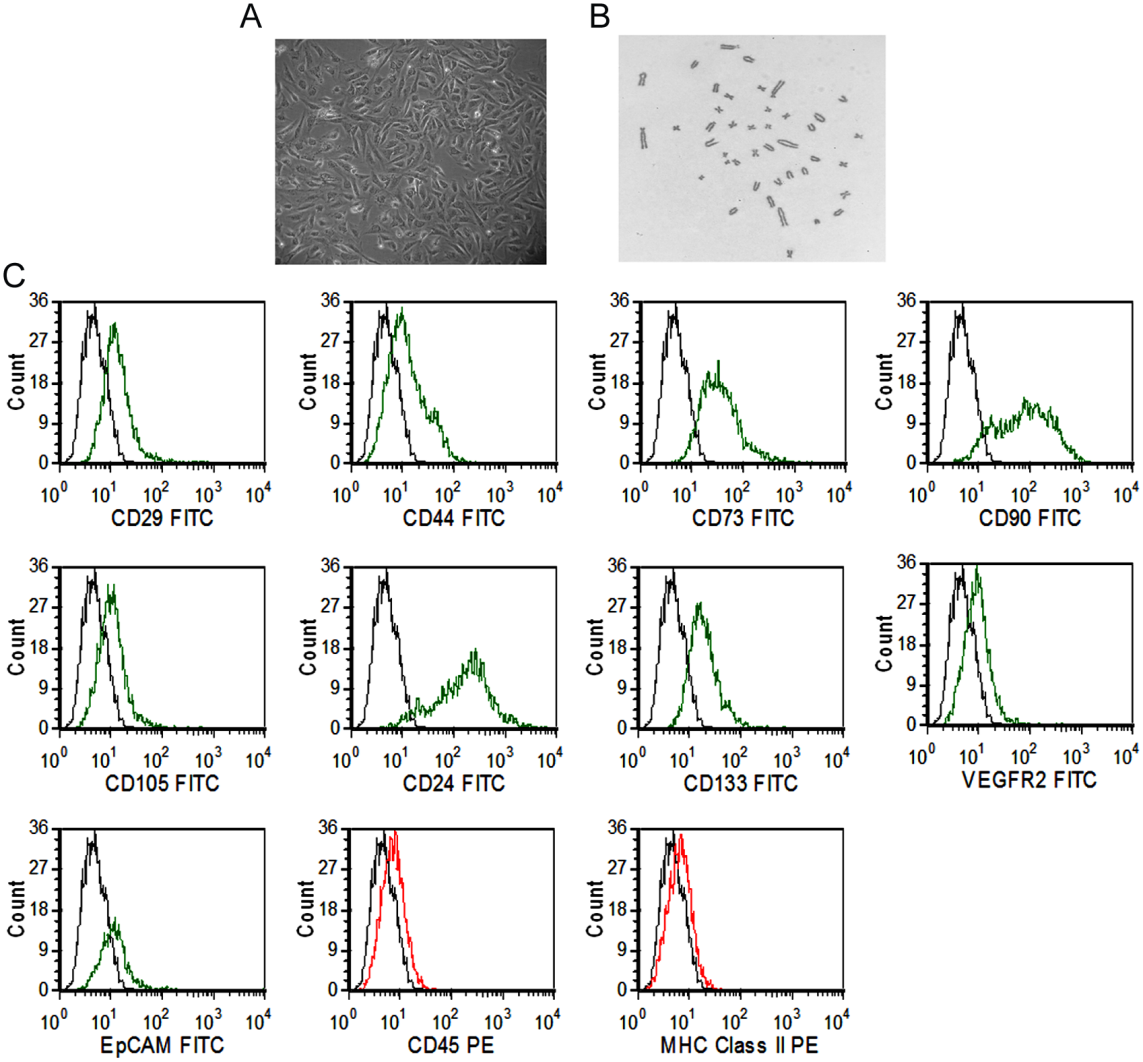
Morphology, karyotype and phenotypic characterization of fetal kidney cells. (A) Representative photomicrograph (10X) of fetal kidney cells in culture, showing spindle-shape and polygonal morphology. (B) The fetal kidney cells showing normal karyotype at 3rd passage (10X). (C) Flow cytometric analysis of fetal kidney cells showing expression of surface markers CD29, CD44, CD73, CD90, CD105, CD24, CD133, VEGFR2, EpCAM, CD45 and MHC class II (green or red lines, detected with FITC- or PE- conjugated antibodies, respectively) with isotype controls (black lines).

The fetal kidney cells isolated from rat fetal kidneys grew in culture as adherent monolayer of cells exhibiting spindle-shaped and polygonal morphology (Fig 1A). The cells were expanded up to 3rd passage and observed to have a consistently normal karyotype (Fig 1B). The fetal kidney cells expressed CD29 (43.87±1.45), CD44 (55.53±1.88), CD73 (58.07±0.95), CD90 (68.07±2.55), CD105 (38.27±1.37), CD24 (60.43±1.54), CD133 (51.93±2.03) and low expression (less than 30%) of VEGFR2 (29.03±1.18), EpCAM (22.20±0.99) with less than 5% expression of CD45 (2.067±0.32) and MHC class II (1.53±0.34) as revealed by their immunophenotypic analysis by flow cytometry (Fig 1C).

### *In vitro* production of renotropic growth factors

**Fig 2:**
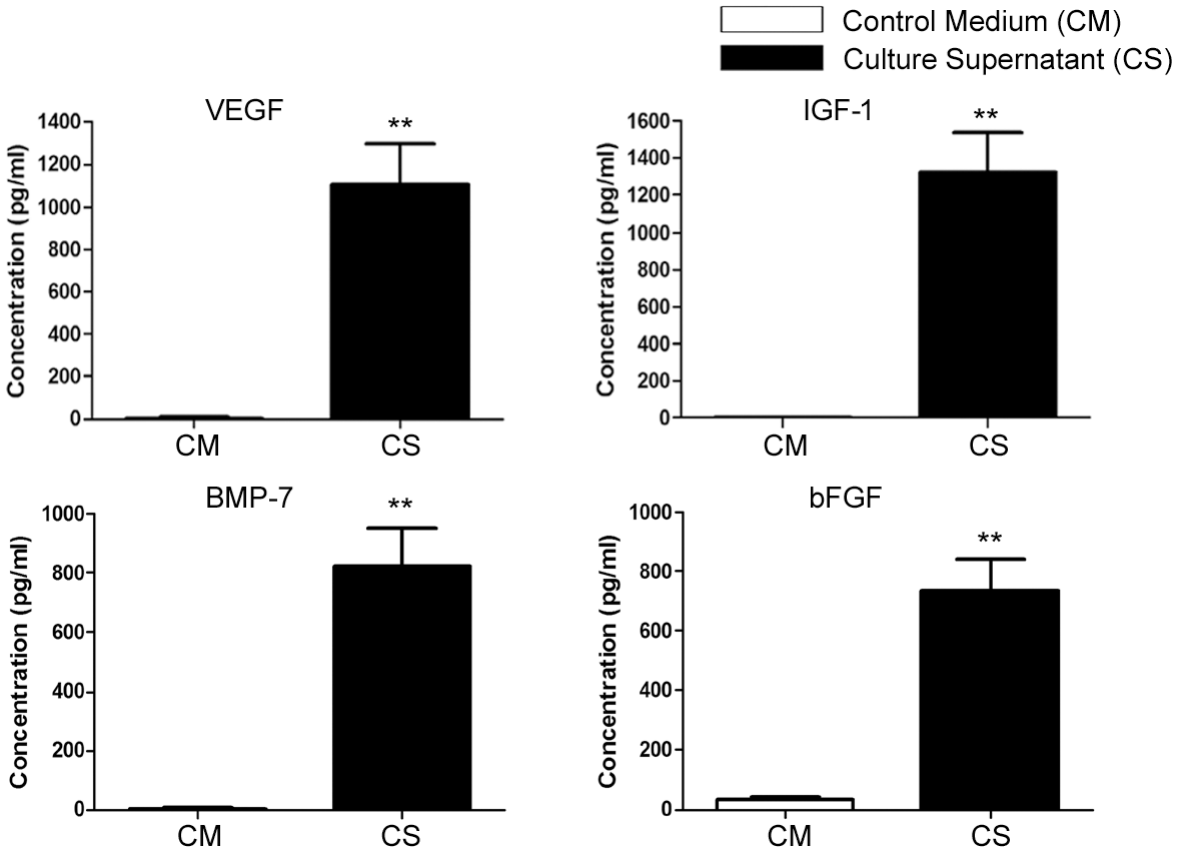
*In vitro* production of renotropic growth factors by fetal kidney cells. The fetal kidney cells significantly produced renotropic growth factors viz. VEGF, IGF-1, BMP-7 and bFGF in their culture supernatant (CS) as compared to fresh culture medium which served as control medium (CM). Values expressed as Mean±SEM. **p<0.01 vs. control medium.

A significantly elevated levels of renotropic growth factors viz. VEGF (1106±189.0 pg/ml), IGF-1 (1322±216.5 pg/ml), BMP-7 (825.8±126.1 pg/ml) and bFGF (737.2±103.1 pg/ml) were observed in culture supernatant as compared to fresh culture medium (Fig 2, p<0.01).

### Effect of fetal kidney cells on renal functions in ARF

**Fig 3:**
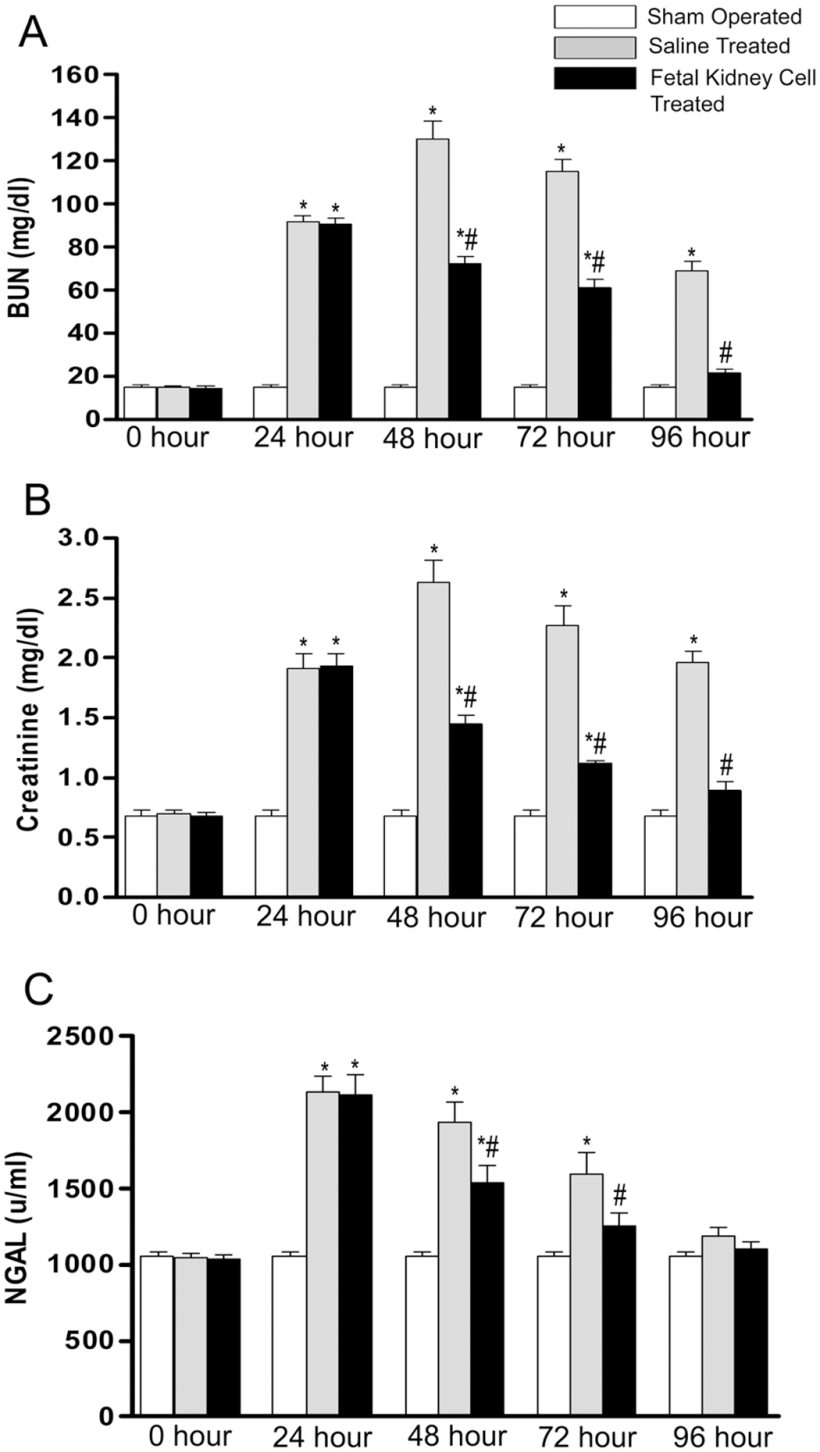
Effects of fetal kidney cells on renal functions in rats with IR ARF. The difference in (A) BUN, (B) serum creatinine and (C) serum NGAL levels in sham operated, saline treated and fetal kidney cells treated groups at different time points (0, 24, 48, 72 and 96 hours after reperfusion). Values expressed Mean±SEM (n = 6). *p<0.05 vs. sham operated group, #p<0.05 vs. saline treated group.

After 24 hours of reperfusion, the rats with IR induced ARF were observed to have a significant increase in the levels of BUN, serum creatinine and serum NGAL as compared to sham operated animals. Among ARF rats one group was given fetal kidney cells while other group was given saline to evaluate effect of fetal kidney cells on renal function. From 48 hours after reperfusion, a consistently significant decrease in the levels of BUN, serum creatinine and NGAL was observed in fetal kidney cells treated rats as compared to the saline treated rats (p<0.05). After 96 hour of reperfusion, the levels of these blood biochemical parameters were comparable to those of sham operated rats (Fig 3A–3C).

### Therapeutic potential of culture supernatant of fetal kidney cells in rat model of ARF

**Fig 4:**
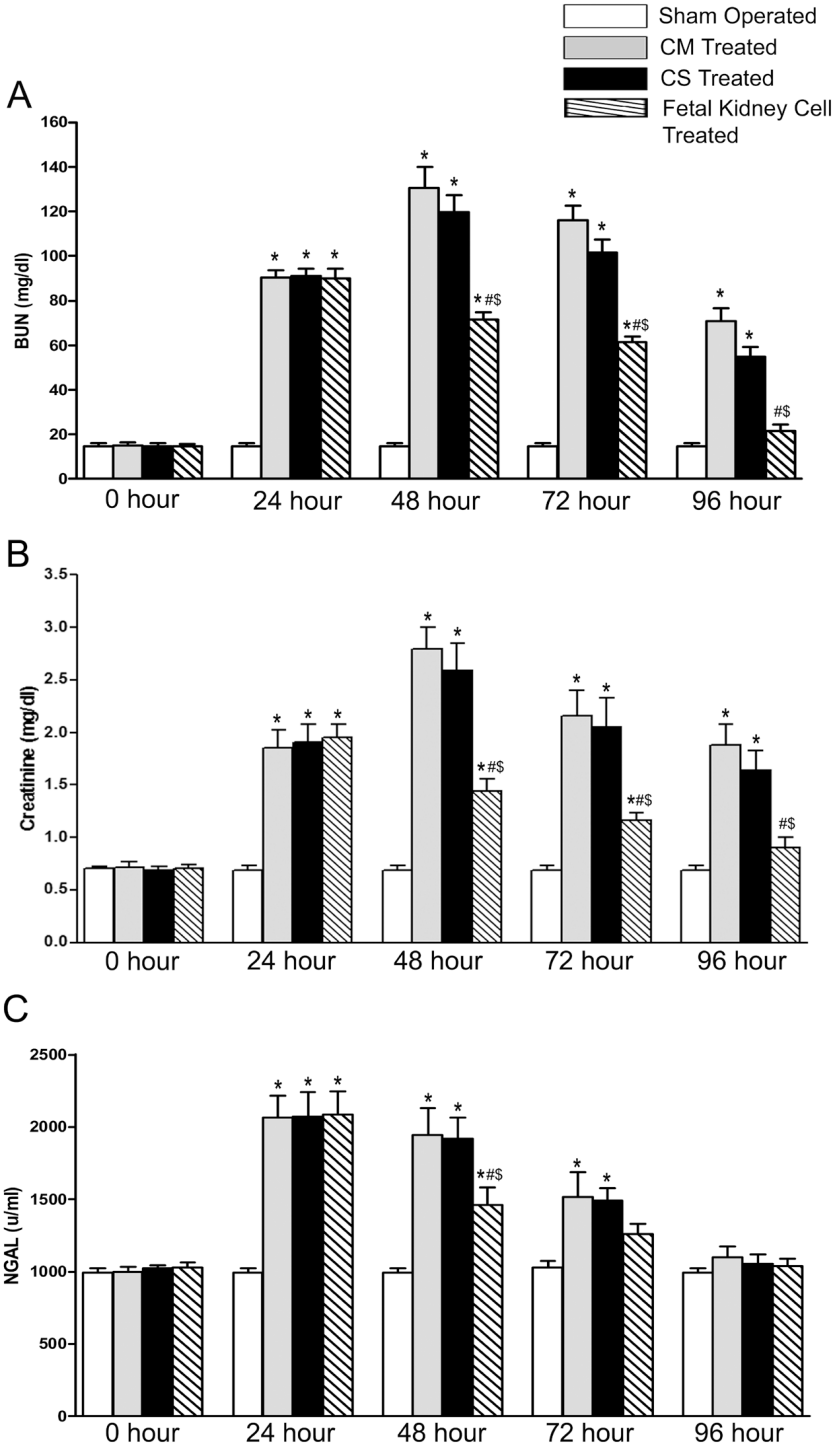
Effects of fetal kidney cells culture supernatant on renal function in rats with ischemic ARF. The difference in (A) BUN, (B) serum creatinine and (C) serum NGAL levels in sham operated, fresh culture medium (CM), fetal kidney cells culture supernatant (CS) and fetal kidney cells treated groups, at different time points (0, 24, 48, 72 and 96 hours after reperfusion). Values expressed Mean±SEM (n = 6). *p<0.05 vs. sham operated group, #p<0.05 vs. fresh culture medium treated and ^$^p<0.05 vs. culture supernatant treated group.

We have found a significant increase in the levels of BUN, serum creatinine and serum NGAL after 24 hours of reperfusion in all animals as compared to sham operated animals (p<0.05). We did not find any significant difference in the levels of BUN, creatinine and NGAL between fresh culture medium and culture supernatant treated rats at 24, 48 and 72 hours after injections. The fetal kidney cells treated animals showed significant decrease in BUN, creatinine and NGAL levels at 48 hours of reperfusion (p<0.05) and became comparable to sham operated group 96 hours after reperfusion. Whereas fresh culture medium and culture supernatant treated group still had significant increase in BUN and creatinine level even after 96 hours of reperfusion as compared to sham operated group (p<0.05; Fig 4A–4C).

### Tracking of PKH26 positive cells at the site of renal injury

**Fig 5:**
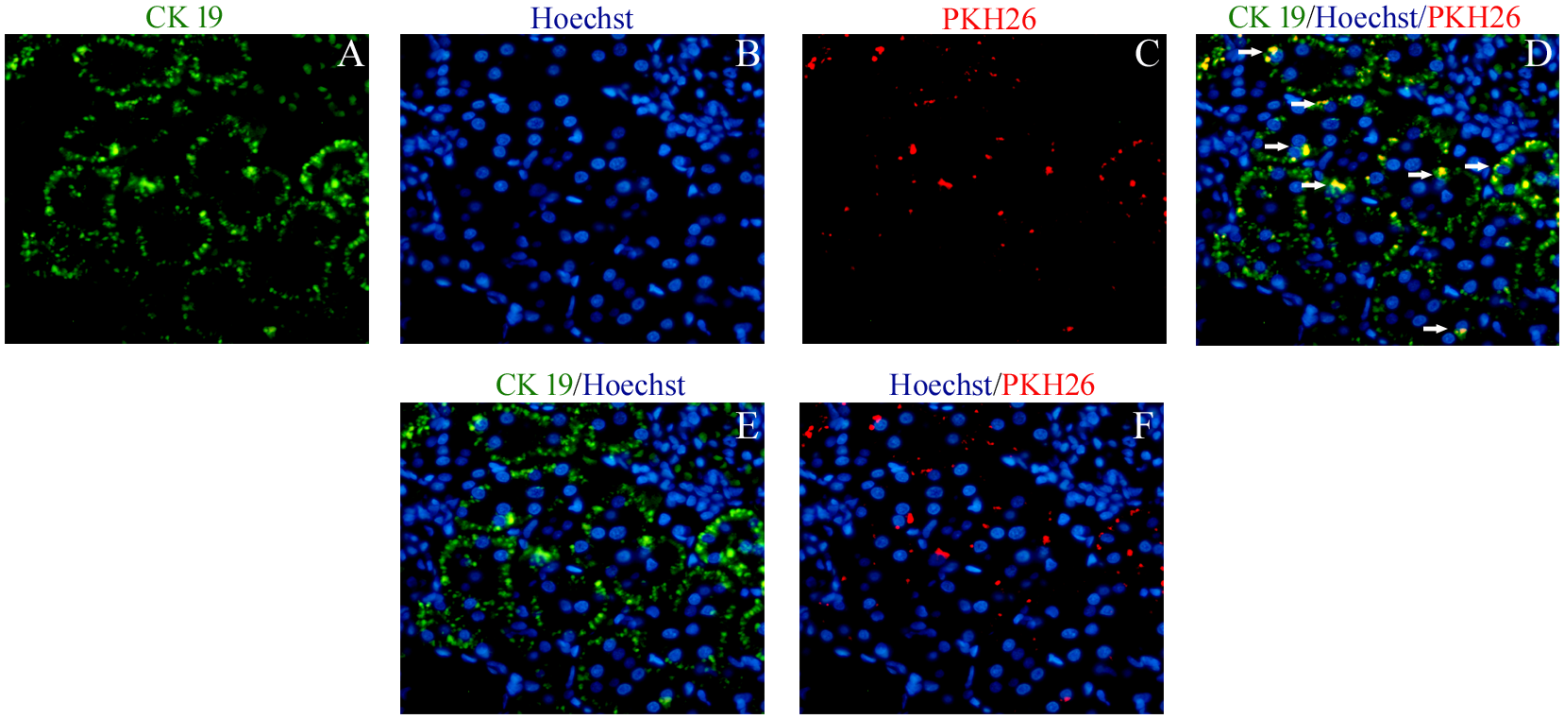
*In vivo* tracking of PKH26 positive cells in IR induced damaged kidney. Representative immunoflourescence photomicrographs (40X) of fetal kidney cells treated kidney showing (A) CK 19, green (B) Hoechst, blue (C) PKH26 labeled cells, red, located in the interstitial spaces and peri-tubular areas of the kidney (D) Overlay of images of (A), (B) and (C). (E) Overlay of images of (A) and (B). (F) Overlay of images of (B) and (C).

The ARF rats infused with PKH 26 labeled fetal kidney cells were sacrificed 72 hours after cell administration and sections of damaged kidneys were examined by fluorescent microscopy. The kidney sections were observed to have 8.28±1.27 PKH26 positive cells/HPF and these cells were found to be localized in the interstitial spaces and peri-tubular areas of the kidney. The tubules stained positive for CK 19 (Fig 5A–5F).

### Effect of fetal kidney cells on ARF progression

**Fig 6:**
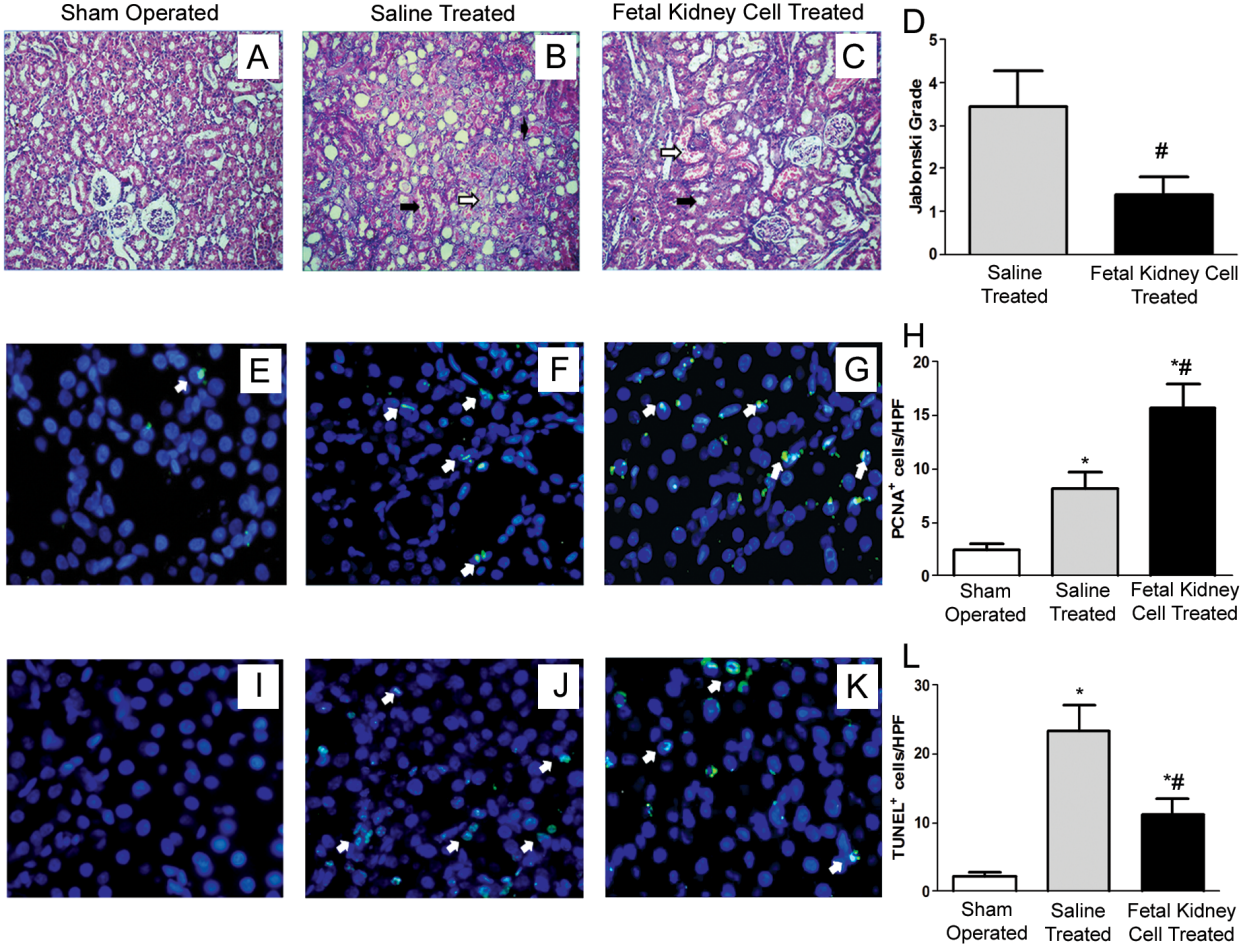
Effects of fetal kidney cells on morphological structure, proliferation and apoptosis of tubular epithelial cells in rats with IR ARF. (A) Kidney section of sham operated animal showing normal architecture of tubules and glomeruli. (B) Kidney section of saline treated animal showing dilated distal convoluted tubule (solid arrow), swollen and necrotic epithelial cells with nuclear changes in the most proximal convoluted tubule (open arrow) and epithelial or hyaline cast material in lumen (solid arrow head). (C) Kidney section of fetal kidney cells treated animal showing signs of recovery as revealed by mild tubular dilatation (open arrow), desquamation of few proximal convoluted tubules and preservation of the integrity of the cellular structure (solid arrow) (20 X). (D) Jablonski grading score of tubular necrosis in saline and fetal kidney cells treated kidneys after 72 hours of fetal kidney cells therapy. (E-G) Representative immunofluorescence photomicrographs (40X) of PCNA staining of kidney sections of sham operated (E), saline treated (F) and fetal kidney cells treated (G) animals. (H) Quantification of PCNA positive cells per HPF. (I-K) Representative immunofluorescence photomicrographs (40X) of TUNEL staining of kidney sections of sham operated (I), saline treated (J) and fetal kidney cells treated (K) animals. (L) Quantification of apoptotic cells per HPF. Values expressed Mean±SEM. (n = 6), *p<0.05 vs. sham operated group, #p<0.05 vs. saline treated group.

Histopathological changes in kidney sections of sham operated rats and saline or fetal kidney cells treated ARF rats were evaluated 96 hours post reperfusion. The sham operated animals showed the normal architecture of tubules and glomeruli (Fig 6A). The kidneys of saline treated rats had extensive tubular necrosis and vacuolization of tubular epithelial cells and their proximal convoluted tubules (PCT) showed a loss of brush border and epithelial cast or hyaline material obliterating their lumens (Fig 6B). In contrast, fetal kidney cells treated kidneys had mild tubular dilation, epithelial desquamation only in few PCT and a low index of cellular damage indicating attenuation of renal injury by infused cells (Fig 6C). Quantitative assessment of renal tubular necrosis after IR injury showed severe tubular necrosis in saline treated as compared to fetal kidney cells treated animals (Jablonski score grade of 3.43±0.35 vs. 1.38±0.16, respectively, p<0.05) (Fig 6D). Immunohistochemical analysis using antibodies against PCNA revealed a significant increase in proliferation of tubular cells in fetal kidney cells as compared to saline treated animals (15.68±0.88 vs. 8.17±0.66 PCNA positive cells per HPF, respectively, p<0.05) (Fig 6E–6H). Moreover, TUNEL assay showed significantly reduced numbers of apoptotic cells in fetal kidney cells as compared to saline treated group (11.25±0.92 vs. 23.25±1.57 TUNEL positive cells per HPF, respectively, p<0.05) (Fig 6I–6L).

### Effect of fetal kidney cell therapy on expression of growth factors and pro- and anti- inflammatory mediators in ARF

**Fig 7:**
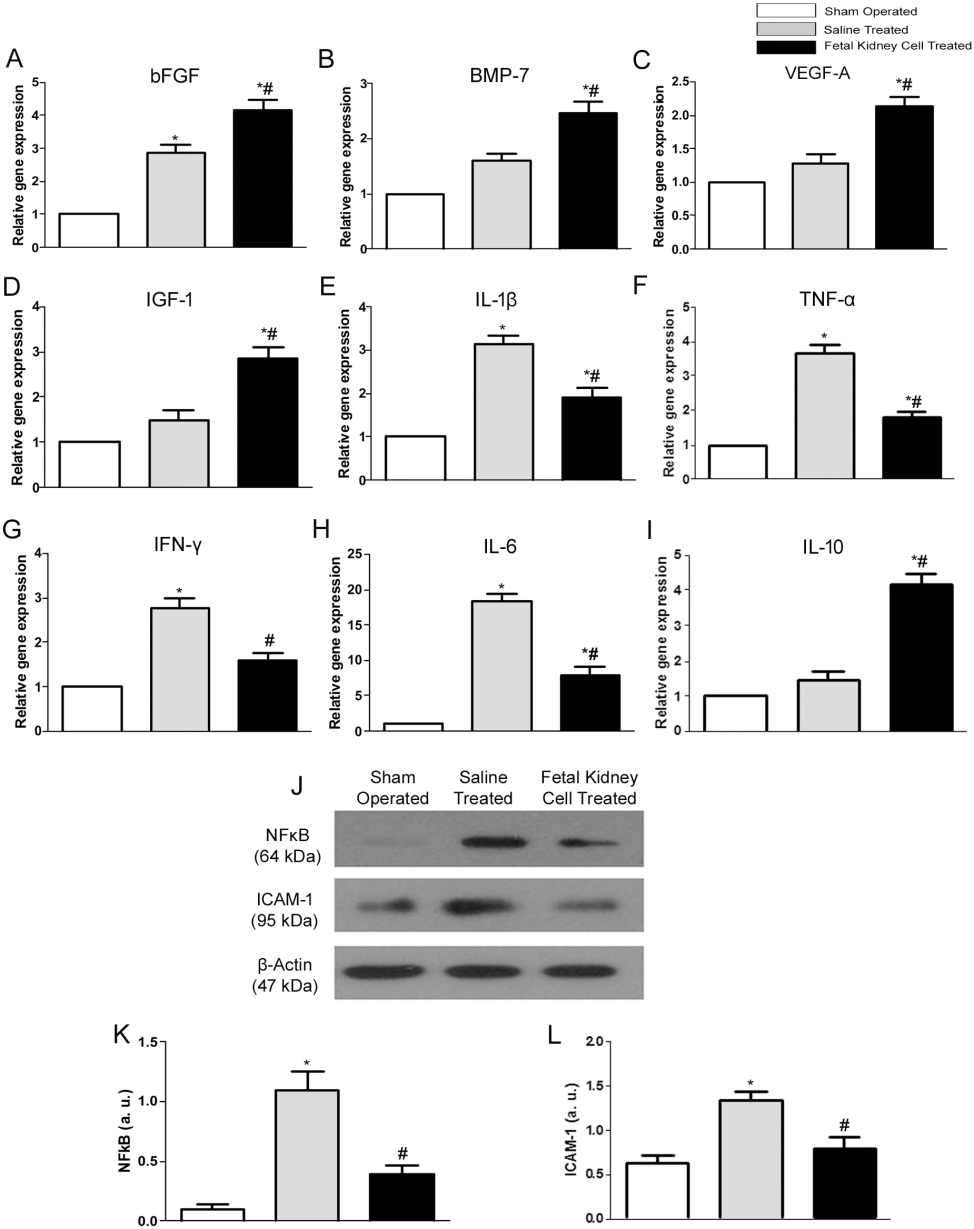
Effects of fetal kidney cells therapy on expression of growth factors and pro- and anti-inflammatory cytokines in rat kidney. (A-I) mRNA expression levels of growth factors viz. bFGF (A), BMP-7 (B), VEGF-A (C) and IGF-1(D), inflammatory cytokines viz. IL-1β (E), TNF-α (F), IFN-γ (G) and IL-6 (H), anti-inflammatory cytokine IL-10 (I). (J) Representative immunoblots showing the expression levels of inflammatory markers viz. NF-kB and ICAM-1 in the kidney tissues of sham operated, saline treated and fetal kidney cells treated groups. (K-L) Bar diagrams showing semi quantitative densitometry of the expression of NFκB and ICAM-1. Comparative gene expression ratio alculated by referring each gene to β-actin as an internal control. Densitometric analysis applied for comparison of relative protein expression and represented in densitometric arbitrary units (a. u.). Values expressed Mean±SEM. *p<0.05 vs. sham operated group. #p<0.05 vs. saline treated group.

The fetal kidney cells treated kidneys showed significantly higher gene expression of growth factors viz. bFGF, BMP-7, VEGF-A and IGF-1 as compared to saline treated and sham operated kidneys (p<0.05) (Fig 7A–7D). The renal expression of genes encoding for the pro-inflammatory cytokines viz. IL-1β, TNF-α, IFN-γ and IL-6 was significantly increased in the saline treated group as compared to sham operated group (p<0.05). However, in fetal kidney cells treated rats, the expression levels of these pro-inflammatory cytokines was significantly decreased, while the expression of anti-inflammatory cytokine IL-10 was significantly increased as compared to saline treated and sham operated groups (p<0.05 for both) (Fig 7E–7I). Furthermore, western blot analysis revealed a significant increase in protein expression of inflammatory biomarkers viz. NFκB and ICAM-1 in saline treated group as compared to the sham operated group (p<0.05). In the fetal kidney cells treated group the expression of these biomarkers were decreased as compared to the saline treated group (p<0.05) (Fig 7J–7L).

### Anti-oxidative and anti-apoptotic effects of fetal kidney cells in ARF

**Fig 8:**
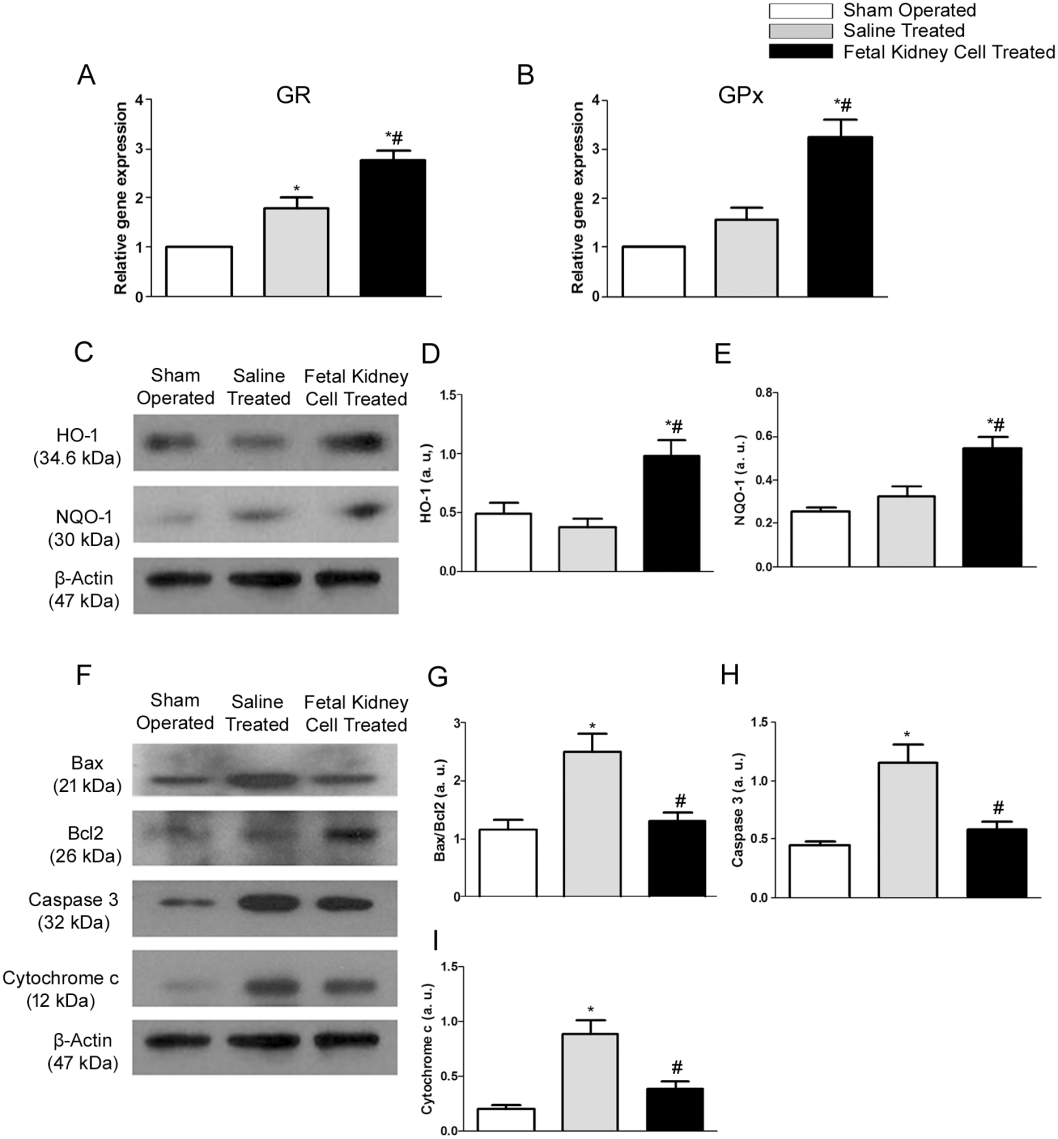
The fetal kidney cells inhibit oxidative stress and apoptosis in kidneys with ischemic injury. (A, B) mRNA expression levels of GR and GPx. (C) Representative immunoblots showing the expression of anti-oxidative biomarkers viz. HO-1 and NQO-1. (D, E) Bar diagrams showing semi quantitative densitometry of expression of the HO-1 and NQO-1. (F) Representative immunoblots showing the expression of apoptosis related biomarkers viz. Bax, Bcl2, caspase 3 and cytochrome c in kidney tissues of sham operated, saline treated and fetal kidney cells treated groups. (G-I) Bar diagrams showing semi quantitative densitometry of a ratio of the expression of Bax/Bcl2 and expression of the caspase 3 and cytochrome c. Comparative gene expression ratio calculated by referring each gene to β-actin as an internal control. Densitometric analysis applied for comparison of relative protein expression and represented in densitometric arbitrary units (a. u.). Values expressed Mean±SEM. *p<0.05 vs. sham operated group. #p<0.05 vs. saline treated group.

Kidneys of animals treated with fetal kidney cells when compared to sham operated or saline treated groups showed significantly higher levels of expression of genes encoding GR and GPx, anti-oxidative enzymes (p<0.05) (Fig 8A and 8B). Treatment with fetal kidney cells also resulted in a significant increase in expression of protein levels of HO-1 and NQO-1, anti-oxidative biomarkers, in comparison to saline treated and sham operated rats (p<0.05) (Fig 8C–8E). A marked increase in Bax/Bcl2 ratio and a significantly higher expression of pro-apoptotic mediators viz. cytochrome c and caspase 3 were observed in saline treated group as compared to sham operated group (p<0.05). After the administration of fetal kidney cells, the up-regulated expression of pro-apoptotic proteins was significantly reduced in comparison to the saline treated group (p<0.05), but comparable to the sham operated group (Fig 8F–8I).

## Discussion

The present study shows that culture expanded fetal kidney cells express mesenchymal and renal progenitor markers. The administration of fetal kidney cells in a rat model of IR induced ARF resulted in the rapid improvement in renal function and attenuation of renal damage. These therapeutic effects of fetal kidney cells were predominantly mediated through their anti-inflammatory, anti-apoptotic and anti-oxidative effects. To the best of our knowledge this represents the first study in literature on the therapeutic effects and mechanism of action of *in vitro* expanded fetal kidney cells in an animal model of ischemic ARF.

Stem/progenitor cells have been reported to exist in both human and rodent fetal kidneys but the biological characteristics and karyotypic stability of culture expanded fetal kidney cells have not been studied [20, 21]. Kim *et al*. in their two independent studies have shown that uncultured rat fetal kidney cells derived from gestation day 14.5 and 17.5 fetuses express mesenchymal markers, and the expression of these markers decline at late gestation stage [22, 23]. These observations corroborate with our data showing expression of mesenchymal markers by fetal kidney cells derived from fetuses of gestation day 16. Kim *et al*. have used de novo isolated fetal kidney cells and have not studied biological characteristics of culture expanded fetal kidney cells [22, 23]. We expanded rat fetal kidney cells up to the 3^rd^ passage and observed that they consistently exhibit a normal karyotype, express mesenchymal (CD29, CD44, CD73, CD90, and CD105) and renal progenitor markers (CD24 and CD133). However, a small fraction (<30%) of these cells also expressed endothelial (VEGFR2) and epithelial (EpCAM) markers. We further evaluated whether fetal kidney cells produce renotropic growth factors *in vitro* and observed presence of elevated levels of VEGF, IGF-1, BMP-7 and bFGF in their culture supernatant. The production of renotropic growth factors by fetal kidney cells have not been reported before.

We infused 2 x 10^6^ PKH26 labeled fetal kidney cells in ARF rats via tail vein and after 72 hours of their infusion, the fluorescent microscopy of the renal tissues showed an interstitial and peri-tubular distribution of the PKH26 positive cells in the injured kidney showing their property to home to injured kidney. Similar to our observation, mesenchymal stem cells (MSC) have been shown to home to injured kidney in rats using magnetic resonance technique [24]. The fetal kidney cells observed by us in the injured kidney may represent only a small fraction of infused cells as a large fraction of intravenously infused cells get trapped into the lungs as recently reported by us using 99m Tc-labeled fetal cardiac MSC [12] and by other groups using other cell types [25]. It has been reported recently that lipophilic dyes including PKH26 may be transferred to host cells mainly due to death of the infused cells and their phagocytosis by resident macrophages [26] and thus the PKH26 positive cells observed by us, may be the infused fetal kidney cells or resident host cells. However, since our observation is after a period of 72 hours of cell infusion when cell death is unlikely, therefore the PKH26 fluorescence present in the kidney at this time point is likely to be from infused cells which have homed to the kidney. It would be advantageous that results obtained from lipophilic dyes are further confirmed using non-lipophylic dyes or other methods such as live imaging techniques.

The infusion of fetal kidney cells in ARF rats resulted in a rapid decrease in the levels of BUN, serum creatinine and NGAL and after 96 hours of IR injury, the levels of these blood biochemical parameters were comparable to those of sham operated rats. We have shown that fetal kidney cells produce several growth factors *in vitro* and hence we also evaluated the therapeutic effect of culture supernatant of these cells in ARF rats. However, we observed that after 72 hours of infusion there was no significant difference in blood levels of BUN, creatinine and NGAL between culture supernatant and fresh culture medium treated ARF rats but fetal kidney cells treated rats showed significant improvement of renal function. Although, we have not used concentrated culture supernatant and thus cannot completely rule out the therapeutic efficacy of culture supernatant. However, no therapeutic effect of culture supernatant in ARF even after its multiple injections in rats indicates that the improvement of renal functions in ARF rats is more likely to be due to fetal kidney cells, which may serve as a continuous source of renotropic factors in the local milieu of the kidney. Similarly other groups have also used multiple injections of unconcentrated culture supernatant in different diseases [17, 27–29]. Histopathological evaluation showed that the fetal kidney cells treated kidneys had attenuation of tubular damage with a quantitative assessment of renal tubular necrosis by Jablonski scoring showed a Jablonski score grade of 1.38±0.16 in fetal kidney cells treated versus 3.43±0.35 in saline treated kidneys (p<0.05). Further, the PCNA assay revealed a significant increase in proliferation of tubular cells and TUNEL assay showed significantly reduced numbers of apoptotic cells in fetal kidney cells as compared to saline treated group (p<0.05). Similar to our observation, Kim *et al*. have shown that transplantation of early gestation stage fetal kidney cells into damaged kidneys in rats resulted in significantly lower BUN levels and significantly greater levels of creatinine in urine as compared to the control rats (no transplantation group). Further, the transplanted rats exhibited kidney tissue reconstitution and the fluorescently labeled transplanted cells were detected in the reconstituted kidney tubules [22]. However, transplantation of fetal kidney cells from a later gestation stage resulted in poor kidney structure formation [23].

After 72 hours of cell infusion, the rapid recovery observed by us in renal functions of fetal kidney cells treated rats may not be due to regeneration of damaged kidney tissues and hence we evaluated whether infused fetal kidney cells mediate rapid recovery of renal function in ARF rats by paracrine mechanisms. We found that kidneys tissues of fetal kidney cells treated ARF rats have a lower mRNA expression of various pro-inflammatory cytokines including IL-1β, TNF-α and IFN-γ and protein expression of other potent inflammatory biomarkers including NFκB and ICAM-1 as well as higher mRNA expression of IL-10, which is a potent anti-inflammatory mediator. These finding suggest that fetal kidney cells could exert rapid renoprotective effects by suppressing ongoing inflammation in the kidneys of ARF rats. In addition we found up-regulated gene expression of various growth factors including bFGF, BMP-7, IGF-1 and VEGF-A as well as proliferation of tubular cells suggesting that fetal kidney cells recruited in the damaged kidney promote renal repair by inducing proliferation of renal tubular and vascular cells. These anti-inflammatory and growth factor mediated paracrine effects of fetal kidney cells observed by us in the present study corroborate with mechanism of renal recovery reported for embryonic stem cell derived mesenchymal progenitors in mice [30]. Furthermore, administration of these growth factors has been reported to limit injury and/or accelerated the recovery in experimental models of ARF [31–33].

Since, oxidative stress and apoptosis represent other important mediators of renal damage in ARF [34, 35], we evaluated whether fetal kidney cells therapy had an impact on the oxidative stress pathway and the intrinsic apoptotic pathway in ARF. We observed that kidneys of fetal kidney cells treated animals had an up-regulated expression of genes of anti-oxidative enzymes viz. GPx and GR and proteins of anti-oxidative biomarkers viz. NQO-1 and HO-1 suggesting that these stem cells could mediate improvement of renal function by preventing renal damage caused by oxidative stress in ischemia induced ARF. These findings of the present study are consistent with the anti-oxidative effect of adipose derived MSC [36] and of antioxidant molecules in protection against ischemic ARF [37, 38]. We also observed that fetal kidney cell therapy results in a decreased apoptosis of tubular epithelial cells in ARF rats and thus wanted to know whether fetal kidney cells regulate expression of various pro- and anti-apoptotic molecules in the kidney tissues. We observed a decreased ratio of expression of Bax/Bcl2 proteins as well as a decreased expression of caspase 3 and cytochrome c proteins in the kidneys of fetal kidney cells treated rats. These findings suggest that fetal kidney cells may inhibit apoptosis of renal tubular cells by up regulating anti-apoptotic molecule Bcl2 and by inhibiting the expression of pro-apoptotic molecules including Bax, caspase 3 and cytochrome c.

In conclusion, our study shows that culture expanded fetal kidney cells express mesenchymal and renal progenitor markers, and rapidly ameliorate ischemic ARF by their anti-inflammatory, anti-oxidative and anti-apoptotic effects. Further studies on the long-term effects of fetal kidney cells in different animal models of ARF would be important for translation of these findings into clinical therapy for the disease.
